# The monitoring possibility of some mammalian cells for zinc concentrations on metallic materials

**DOI:** 10.1007/s10616-012-9433-6

**Published:** 2012-02-14

**Authors:** Akiko Ogawa, Naoaki Okuda, Katsuya Hio, Hideyuki Kanematsu, Hidekazu Tamauchi

**Affiliations:** 1 Department of Chemistry and Biochemistry, Suzuka National College of Technology, Suzuka, 510-0243 Japan; 2 Department of Materials Science and Engineering, Suzuka National College of Technology, Suzuka, 510-0243 Japan; 3 Mie Prefecture Industrial Research Institute Metal Science Branch, Kuwana, 511-0937 Japan; 4 Kitasato University School of Medicine, Sagamihara, 225-8555 Japan

**Keywords:** Colony-forming assay, V79 cells, Corrosion, Tin-zinc alloys

## Abstract

Zinc plating is widely used to protect steels against corrosion. However, the possibility of a high environmental risk for zinc has been recently discussed among advanced countries and more environmentally-friendly substitutes are required urgently. Therefore, monitoring zinc concentration changes on metallic materials such as steel is very important. We chose to measure zinc concentration changes in some mammalian cells and confirmed that V79 cells were highly sensitive to changes in zinc concentrations. In this study, the following process was applied to the proprietary production for tin-zinc alloy films on steel using V79 cells. Specimens were immersed in PBS to produce extracts. Zinc concentrations in the extracts almost corresponded to zinc concentrations on steel surfaces. When extracts were added to a V79 cell culture, colony formation was inhibited, and inhibition increased with increases in zinc concentrations. Changes in zinc concentrations on steel surfaces with heat treatment could be monitored relatively well by V79 cells, even though the results were still semi-quantitative.

## Introduction

Zinc has been used as an important plating metal element for steel materials as it shows high corrosion resistance in various environments such as air and neutral aqueous environments (Williams 2000; Simon-Hertich et al. 2001; Hadley et al. 2002). However, the possibility of a high environmental risk for zinc has been recently discussed among advanced countries. As a result, alloy plating of zinc with some other metals was proposed as a substitute. Alloy elements are environmentally-friendly and alloying should decrease harm to the environment. Such an investigation and development will be more common in the near future.

One of the authors proposed a new tin-zinc alloy production method to alleviate the environmental burden caused by the presence of zinc (Kanematsu et al. 2001a, b, 2002a, b). Although the production method requires precise information about component changes on steel surfaces, sometimes instrumental analyses cannot detect these changes.

As such, analytical methods to identify metal components on steel surfaces qualitatively and quantitatively are urgently required. Even though there are already many analytical methods using instruments, and the accuracy has been increasing year by year, more direct, user-friendly, understandable, and inexpensive methods are desirable. Some new proposals for such a purpose may be possible. With these new innovative methods, changes in metallic components on steel surfaces could be detected rapidly and precisely.

We investigated the biological reaction of mammalian cells with metal elements such as zinc and steel and found that some cells were sensitive to changes in metal ion concentrations in aqueous solution (Kanematsu et al. 2008; Ogawa et al. 2009).

In this study, we have explored the possible use of biological assays to evaluate the physical properties of different tin-zinc alloys. We used a colony-forming assay on extracts of some tin-zinc alloys produced by a proprietary method. We also analyzed the surface conditions of tin-zinc alloys and determined the metal concentration of extracts prepared from these alloys. We then considered the correlation between the results of the biological assay and the surface conditions of the tin-zinc alloys. Finally, we discussed the application possibilities of mammalian cells to monitoring zinc concentration changes on steel surfaces.

## Materials and methods

### Materials

Carbon steel (JIS SS400) was used as the substrate for plated specimens. A 1 μm zinc coating or tin coating was electroplated onto carbon steel by an outside supplier. Additional tin coating was carried out on some of this zinc plated steel using a radio frequency magnetron spattering machine (Beamtron, Ibaraki, Japan). The thickness of the tin coating was about 0.135 μm. To make a tin-zinc alloy, tin zinc layered SS400 steels were heated for 1 h at three different temperatures: 250, 350, or 450 °C to produce SnZn-250, SnZn-350, and SnZn-450 specimens, respectively. An unheated tin zinc layered specimen was referred to as SnZn-0. All specimens were cut into small coupons (1 cm^2^) with a shearing machine (Komatsu Industry, Ishikawa, Japan) and the cut edges, which were not covered with tin zinc coating, were painted with a white marker pen to protect them from corrosion. Coupons were not polished using abrasives so that the surface layer would not be removed. Instead, specimens were sterilized using two different processes (autoclave sterilization at 121 °C for 15 min and a wiping-out process using alcohol) and were then used in the following experiments.

### Cell and culture conditions

Firstly, mammalian cells appropriate for the purpose of this study had to be carefully selected. Cells had to satisfy the following three conditions. Cells had to be easily available, had to be easy to culture, and finally, some of its functions had to be useful for the evaluation. After a review of the literature, we found two kinds of mammalian cells. One was the V79 cell derived from Chinese hamster lung fibroblasts. V79 cells have been used for many carcinogen assays because its characteristics include a remarkable tendency to form strong colonies, a relatively short doubling time, and sensitivity to mutagenic components (Kuroki et al. 1980). Nowadays, it is used for the toxicity test of medical equipment (ISO 10993-5:2009(E)). The other cell selected was the HepG2 cell derived from human hepatomas. These cells have relatively higher liver functions such as albumin production and drug metabolizing activities. Nowadays, it is used for hybrid-type artificial livers and many drug assays (Hoekstra and Chamuleau 2002).

V79 cells were obtained from the Health Science Research Resources Bank (JCRB0603, Osaka, Japan). Cells were maintained in a humidified atmosphere, 5% CO_2_-95% air at 36.5 °C, and were grown in minimum essential medium (MEM: Invitrogen, Grand Island/NY, USA) containing 5% fetal bovine serum (FBS: Biowest, Nuaillé, France). For subculturing, V79 cells were removed enzymatically (0.1% trypsin–EDTA), split 1:8–10, and subcultured in 35 mm diameter petri dishes (Sumitomo Bakelite, Tokyo, Japan). HepG2 cells were obtained from the RIKEN Bioresource Center (RCB1886, Tsukuba, Japan). Cells were maintained in a humidified atmosphere, 5% CO_2_-95% air at 36.5 °C, and were grown in Dulbecco’s modified Eagle medium (DMEM: Nissui, Tokyo, Japan) containing 5% FBS, 0.2% sodium bicarbonate, 10 mM HEPES (Sigma-Aldrich, St. Louis, MO, USA), and 2 mM glutamine. For subculturing, HepG2 cells were removed enzymatically (0.1% trypsin–EDTA), split 1:3–4, and subcultured in 90 mm diameter petri dishes (Sumitomo Bakelite, Tokyo, Japan).

### Elution from metal samples

Cleaned metal specimens were soaked with 70% ethanol overnight. Each sterilized metal specimen was placed in a sterilized 15 ml plastic tube (Sumitomo Bakelite, Tokyo, Japan) containing 10 ml of phosphate buffered saline (PBS). Tubes were heated to 36.5 °C in a water bath for 22–24 h. Metal specimens were then removed from the tubes to halt the extraction process. The liquid in these tubes was examined in the colony forming assay.

### Growth assay

HepG2 cells were seeded on 24-well plastic cell culture plates (Sumitomo Bakelite) at 104,600 cells/well and were pre-cultured for 1 day. The next day, zinc sulfate solution (Wako Pure Chemical Industries) or PBS (100 μl/well) was added to each well of the culture plate, which also contained 500 μl/well of 5% FBS-DMEM, and cells were cultured for 3 more days. On day 4, viable and dead cell numbers were determined by the trypan blue exclusion method using an improved Neubauer hemocytometer (Erma, Tokyo, Japan).

### Colony forming assay

V79 cells were seeded on 12-well plastic cell culture plates (Becton–Dickinson, Franklin Lakes/ NJ, USA) and precultured for 1 day. The next day, 400 μl of zinc sulfate solution or 400 μl of extract/PBS was added to each well of the culture plate, which also contained 1 ml/well of 5% FBS-MEM, and cells were cultured for several more days. After that, the growth medium was removed and the colonies that had formed were washed twice with PBS. Next, they were fixed with 100% methanol (Wako Pure Chemical Industries, Osaka, Japan) for 1 min and stained with Giemsa solution (Wako Pure Chemical Industries). Finally, stained colonies were rinsed with distilled water several times and counted visually.

### Measurement of metal concentrations in extracted solutions

The metal concentrations of each extract were determined using an Inductively Coupled Plasma Atomic Emission Spectrometer (Shimadzu, Kyoto, Japan).

### Surface elements analysis of metals

The surface structures of all tin-zinc alloys were investigated by X-ray diffraction analysis. The X-ray diffractometer used in this series of analyses was a RINT 2100 (Rigaku, Tokyo, Japan), equipped with a copper X-ray tube. The X-ray voltage was 40 kV and the current was 20 mA. The diffraction angle was varied from 20 to 100° and the scan rate was 2°/min.

## Results and discussion

**Fig. 1:**
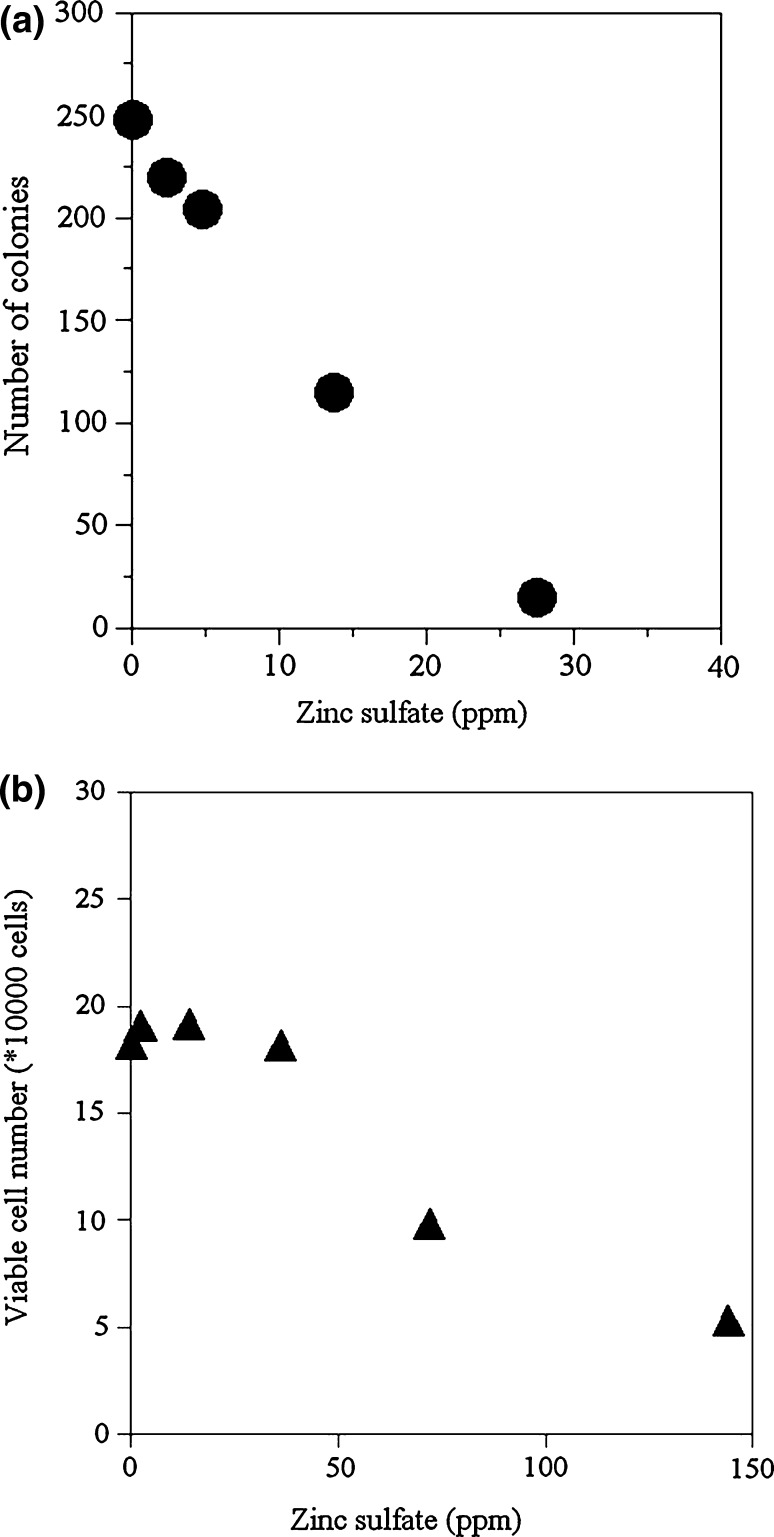
The effect of zinc sulfate on cell growth. V79 cells were tested for a colony forming assay (**a**) and HepG2 cells were used for a growth assay (**b**). The *vertical axis* corresponds to the concentration of zinc sulfate. **a** The *horizontal axis* corresponds to the number of colonies (n = 2). The relationship between the concentration of zinc sulfate: [x] and the number of colonies: [y] are correlated. The regression equation is linear: [y] = −8.48 [x] + 242 and the correlation coefficient is 0.99. **b** The *horizontal axis* corresponds to the number of viable cells (n = 2)

Firstly, the sensitivity of chosen mammalian cells for zinc was investigated by measuring changes in cell growth with zinc sulfate. Results are shown in Fig. 1a and b. Figure 1a corresponds to the results of V79 cells and Fig. 1b to that of HepG2 cells. Decreasing the number of colonies or viable cell numbers obviously related to arrested cell growth or cell death by zinc ions in the culture solution, and the index could be used to measure zinc ions in the solution (Fig. 1a, b). As the figures clearly show, the sensitivity of V79 cells to zinc ions in the solution was much higher than that of HepG2 cells. The V79 cell line was then chosen for the following investigations.

**Fig. 2:**
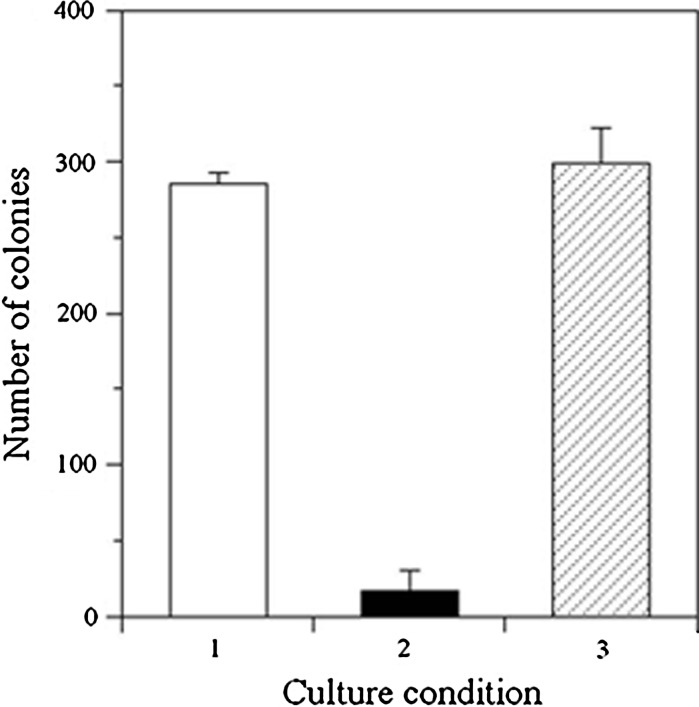
The effect of zinc coating and tin coating on colony formation. Each *column* indicates the number of colonies formed in the culture condition (n = 3) where V79 cells were cultured in medium with the extract of SS400 (*1*), zinc coated SS400 (*2*), or tin coated SS400 (*3*)

Since the purpose of this study was to measure changes in zinc concentrations on metal surfaces using mammalian cells, specimens were immersed into PBS to extract metallic ion components from solid surfaces. As for the three kinds of specimens (SS400, zinc coated specimen, tin coated specimen), extracted solutions were produced and were added to a V79 cell culture. Colony formation was inhibited in the extract of the zinc coated specimen, while colonies formed without inhibition in the extracts of the SS400 or tin coated specimens (Fig. 2). These results indicate that the extract of zinc coated steel had enough metal ions to inhibit colony formation while the extract of tin coated steel did not. The concentrations of various metallic ions in the extracts were measured by inductively-coupled plasma atomic emission spectrometry (ICP-AES) and were summarized in Table 1. As shown in Fig. 2, even though the concentration of iron ions in the SS400 extract was relatively large, colony formation was not inhibited, suggesting that V79 cells were not sensitive to iron ions. On the other hand, colony formation was inhibited significantly in the zinc coated extract, even though the zinc concentration was very low (Table 1), indicating the high sensitivity of V79 cells to zinc concentrations in the solution. For the tin coated extract, colony formation was the same as that in the SS400 extract. Although the tin concentration was also very low in the extract, V79 cells were not sensitive to these concentrations. These results suggest that V79 cells may be used to monitor only zinc ions in extracts.

**Table 1:** Metal concentration of the extracts of zinc or tin coated SS400

	Fe (ppm)	Sn (ppm)	Zn (ppm)
SS400	0.34	ND	ND
Sn-coating	0.05	<0.01	<0.001
Zn-coating	<0.01	ND	0.09

*ND* means not detected

Since V79 cells reacted sensitively to zinc ions in the extract, we applied the process to tin-zinc alloy films on steel produced by our novel plating process. In this process, stacked zinc and tin single phases by various surface coating processes are heated to around melting temperatures to produce alloy phases. Since V79 cells could respond sensitively to zinc ion concentrations in the extracted solution, as already described, we attempted to apply these cells to alloy film systems and investigated if V79 cells could monitor the phase change in tin-zinc alloy films on steel surfaces.

**Fig. 3:**
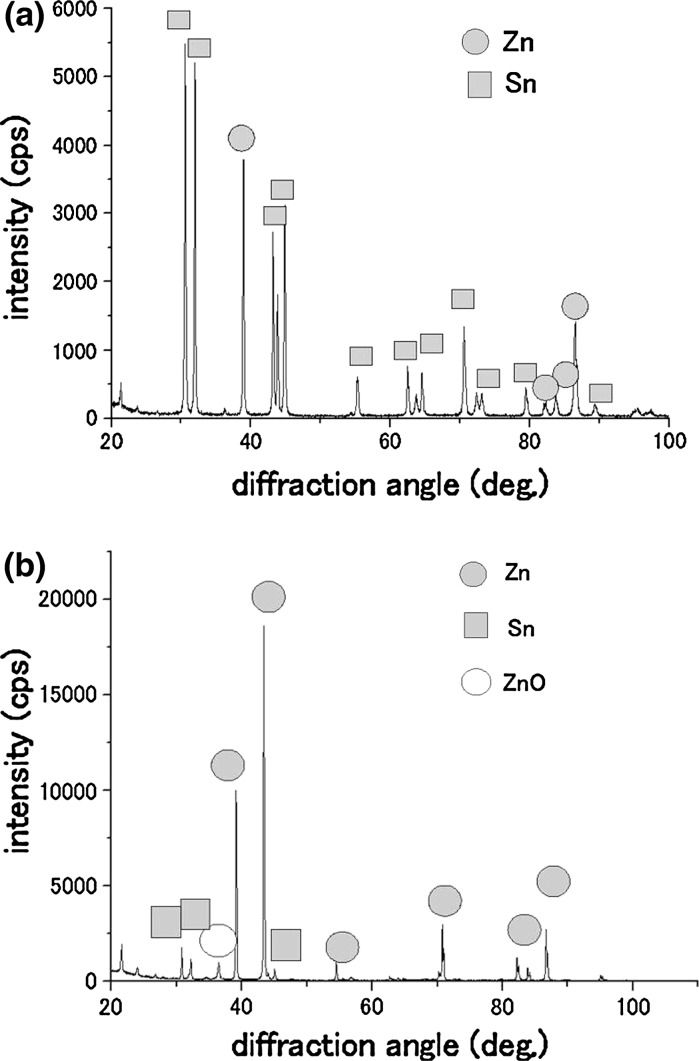
X-ray diffraction analysis of the surface of tin-zinc coated steels. The *vertical axis* corresponds to X-ray intensities and the *horizontal axis* to diffraction angles. *Closed circles* indicate zinc, *open circles* indicate zinc oxide, and *closed squares* indicate tin. **a** Before heat treatment; **b** Heat treated at 250 °C

Figure 3a shows an example X-ray analysis for a specimen before heat treatment. The vertical axis corresponds to the intensity of the X-ray and the horizontal one to the diffraction angle. Each peak in the figure could be identified as zinc or tin. The iron peak was not detected, since the surface layers (about 1 μm thickness) protected the substrate from X-ray irradiation. When the heat treatment temperature was elevated, peaks for zinc oxide appeared as shown in Fig. 3b. In Fig. 3b, the specimen was heated to 250 °C and the zinc component was oxidized on the specimen’s surface to some extent. For X-ray diffraction peaks at each temperature, peak ratios were calculated and compared among three phases, tin, zinc, and zinc oxide. Results were summarized in Table 2.

**Table 2:** Surface conditions of zinc coated SS400 and tin-zinc alloys

Alloy	Sn (%)	Zn (%)	ZnO (%)
Zinc coated SS400	0	100	0
SnZn-0	73	27	0
SnZn-250	30	60	10
SnZn-350	0	62	38
SnZn-450	0	18	82

The peak ratio indicates the concentration among phases on the surface semi-quantitatively, since the specimen’s surface was irradiated by X-ray. As shown in Table 2, heat treatments at 250 and 350 °C increased the zinc ratio in the vicinity of the specimens’ surfaces, while the tin ratio decreased. Zinc atoms under the surface of the tin layer diffused into the upper tin layer by heating, and tin diffused into the lower zinc layer to produce alloy films. However, at 450 °C, zinc was consumed to form zinc oxide and as a result, the concentration of zinc on the surface decreased, while the concentration of zinc oxide increased. According to these results, zinc concentrations decreased in the following order.

1 $$ {\text{Zinc plated SS}}400 > {\text{SnZn-}}350 > {\text{SnZn-}}250 > {\text{SnZn-}}0 > {\text{SnZn-}}450 $$

In this experiment, pure zinc coated specimens were used for controls. SnZn-0 refers to the specimen before heat treatment and SnZn-X to specimens heated at X °C.

When these specimens were immersed in PBS, zinc, tin, and iron ions dissolved into the solution, according to each surface concentration. Concentrations in the extracts measured by ICP-AES were summarized in Table 3. Zinc concentrations decreased in the following order.

2 $$ {\text{Zinc plated SS}}400 > {\text{SnZn-}}350 > {\text{SnZn-}}0 > {\text{SnZn-}}250 > {\text{SnZn-}}450 $$

**Table 3:** Metal concentrations of the extracts of zinc coated SS400 and tin-zinc alloys

Alloy	Zn (ppm)	Sn (ppm)	Fe (ppm)
Zinc coated SS400	13.0	ND	0.05
SnZn-0	5.1	6.9	0.02
SnZn-250	4.6	2.5	0.03
SnZn-350	6.8	1.2	0.04
SnZn-450	2.0	0.08	0.3

*ND* means not detected

**Fig. 4:**
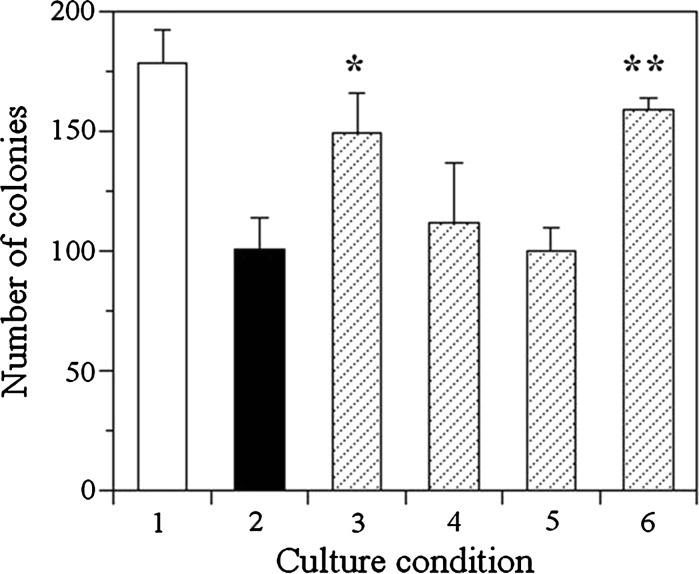
The number of colonies formed in the medium with the extract of each alloy. Each *column* indicates the number of colonies formed in the culture condition (n = 3) where V79 cells were cultured in medium with the extract of zinc coated SS400 (*2*), SnZn-0 (*3*), SnZn-250 (*4*), SnZn-350 (*5*), or SnZn-450 (*6*), with only PBS (*1*). Significant differences were confirmed statistically: Student’s *t* test. *p < 0.05; **p < 0.005

These extracts were added to cells and colony formations were observed. The results were shown in Fig. 4. No. 1 (No) corresponds to the result of controls, where PBS was added to cells as a reference. Other connotations were the same as in Table 3. In this figure, effects on colony formation decreased in the following order.

3 $$ {\text{Zinc plated SS}}400 \, = {\text{ SnZn-}}350 > {\text{SnZn-}}250 > {\text{SnZn-}}0 > {\text{SnZn-}}450 $$

As discussed above, it was assumed that the extent of inhibiting colony formation corresponded to the zinc concentration on the surfaces of specimens. According to our speculations, order (3) (as established using colony forming assay and presented in Fig. 4) should be the same as order (1) (as established using X-ray diffraction analysis and presented in Table 2) and order (2) (as established using ICP-AES and presented in Table 3). If the result of SnZn-0 were eliminated, all of these results would be equal. However, order (2) was the only exception. The reason could be explained as follows.

Pure zinc plated specimens in Tables 1 and 3 showed different zinc concentrations, respectively, which may be attributed to different sterilization processes. The zinc plated specimen in Table 1 was sterilized at 121 °C for 15 min in the autoclave, while that in Table 3 was sterilized at room temperature by just wiping-out with alcohol. The former process may accelerate the dense and thin hydroxide film of zinc on the specimen’s surface, which retarded the dissolution of zinc in the following process. However, the same alcohol sterilization was applied to all of the tin-zinc alloy film specimens. Therefore, it did not affect the comparison of results.

**Fig. 5:**
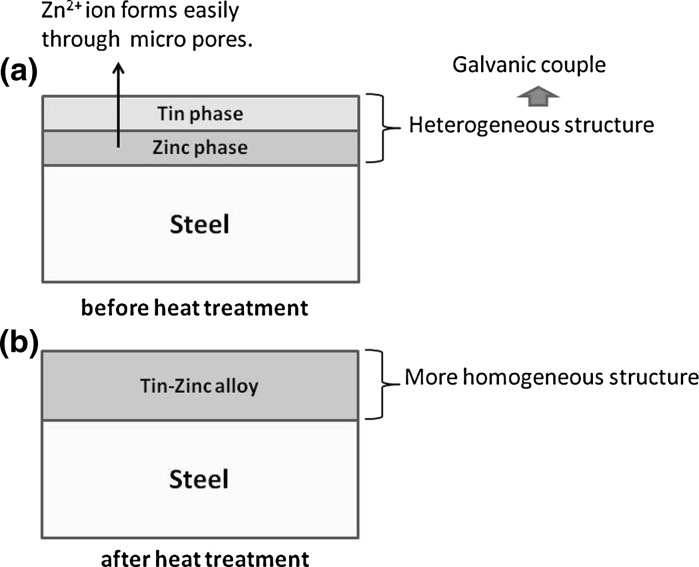
Schematic illustration of cross sections for specimens used in this study

The surface structure of SnZn-0 could be described schematically as shown in Fig. 5a. The surface film was composed of tin and zinc layers which were stacked separately and individually. On the other hand, the surface layers for other specimens were shown in Fig. 5b schematically. For the latter, tin and zinc layers were alloyed to some extent. From an electrochemical viewpoint, the former was heterogeneous and unstable by the galvanic effect. Therefore, the dissolution of zinc could be accelerated. On the other hand, the latter alloy films were homogeneous and electrochemical dissolution was lower than that in the stacked tin-zinc layers specimen. Therefore, only the position of SnZn-0 in order (2) was different from that of the other orders. This result suggests that V79 cells could be used to monitor changes in zinc concentrations on alloy filmed specimens.

**Fig. 6:**
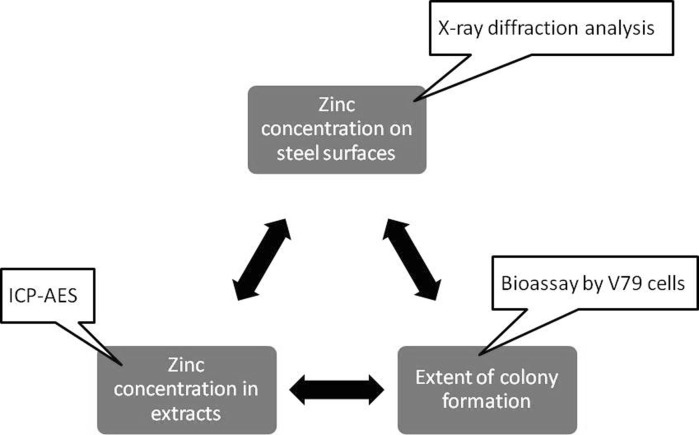
Schematic concept for monitoring zinc concentrations on steel surfaces using V79 cells. Each *balloon* indicates the analytical method of the target. *Double-pointed arrows* indicate the proportional relationship between the two targets

To summarize all of the results mentioned above, V79 cells could be used to monitor changes in zinc concentrations on steel surfaces, as long as measurements were related only to tin-zinc alloy film specimens. The monitoring process is shown in Fig. 6 schematically. Changes in zinc concentrations on steel surfaces could be reflected by zinc concentrations in the extracts. V79 cells will “measure” changes in zinc concentrations in extracts. Even though these experimental results were still semi-quantitative, the precision and accuracy could be improved in the future. If so, V79 cells will be a good sensor for zinc concentration changes on steel surfaces in the future.

## Conclusions

Measuring zinc concentration changes on steel surfaces was carried out using mammalian cells. V79 and HepG2 cells were selected and their sensitivity to zinc, tin, and iron was investigated. We confirmed that V79 cells could be used as a monitor for changes in zinc concentrations on steel surfaces. The process was applied to proprietary alloy film steels, tin-zinc alloy film steels. Specimens were immersed into PBS to produce extracts. Zinc concentrations in the extracts almost corresponded to those on steel surfaces. When extracts were added to a V79 cell culture, colony formation was inhibited, and this inhibition increased with increasing zinc concentrations. These results suggest that V79 cells could be used to monitor changes in zinc concentrations on steel surfaces.
